# Accelerated biological aging based on DNA methylation clocks is a predictor of stroke occurrence: a systematic review and meta-analysis

**DOI:** 10.3389/fneur.2025.1640853

**Published:** 2025-11-06

**Authors:** Jiacai Feng, Xingyu Huang, Rongqing Wu, Guohui Ding, Ming Liu, Renli Deng

**Affiliations:** 1 Nursing Department, The Affiliated Hospital of Zunyi Medical University, Zunyi, Guizhou, China; 2 International Human Phenome Institutes, Shanghai, China; 3 Peking University Health Centre-Macao Polytechnic University, Macau, Macao SAR, China

**Keywords:** DNA methylation, stroke, biological age, epigenetic age acceleration, meta-analysis

## Abstract

**Background:**

Although traditional vascular risk factors, such as hypertension and diabetes, are incorporated into stroke risk prediction models, a significant proportion of stroke events remain unexplained by these models. Increasing evidence suggests that accelerated biological aging, as measured by DNA methylation clocks, may reflect reduced organ function and heightened susceptibility to disease. However, the relationship between epigenetic age acceleration (EAA) and stroke risk remains poorly understood, with limited comprehensive synthesis of the available evidence.

**Methods:**

We conducted a systematic search of PubMed, Embase, Web of Science, and Cochrane Library databases (up to January 10, 2025) for observational studies examining the relationship between DNA methylation-derived EAA and stroke risk. The study protocol was registered with PROSPERO (CRD420251010621).

**Results:**

Thirteen studies met the inclusion criteria. Random-effects meta-analysis revealed a significant positive association between accelerated biological aging and stroke risk (OR = 1.16, 95% CI 1.13–1.19, *I*^2^ = 98.9%, *p <* 0.001). Stratified analysis by stroke event demonstrated a stronger association with incident stroke (OR = 1.28, 95% CI 1.25–1.35, *I*^2^ = 92.6%, *p* = 0.001) compared to stroke recurrence (OR = 1.11, 95% CI 1.06–1.16, *I*^2^ = 63.6%, *p* = 0.041). Sensitivity analyses confirmed the robustness of these findings.

**Conclusion:**

DNA methylation-derived measures of accelerated biological aging are robust predictors of stroke. These findings provide new insights into stroke risk assessment and emphasize potential biomarkers for early detection and prevention. Further large-scale prospective studies are needed to validate these associations and examine the role of additional modifying factors.

## 1 Introduction

Stroke, ranked as the second leading cause of death globally and the primary etiology of adult disability, has seen its pathogenesis and precise risk factor identification remain a central focus in neuroscience research (1, 2). Epidemiologically, one in four individuals will experience a stroke during their lifetime, with one-third of survivors developing long-term disabilities (3). Although improved secondary prevention strategies have reduced stroke incidence in recent years, its prevalence continues to rise due to increasing life expectancy.

Current clinical risk assessment models incorporate traditional factors such as hypertension, diabetes, and smoking. However, a proportion of stroke events remain unexplained by existing models, suggesting undiscovered biological mechanisms driving stroke event (4, 5). In this context, epigenetic regulation—a critical bridge between genotype and phenotype—has demonstrated groundbreaking progress in aging-related disease research. Notably, deoxyribonucleic acid (DNA) methylation-based epigenetic clocks quantify methylation variations at specific cytosine-guanine dinucleotides (CpG) sites, providing an innovative tool to assess biological aging rates (6). Studies indicate that epigenetic age acceleration (the deviation between DNA methylation age and chronological age) may reflect pathophysiological processes like organ functional decline and inflammatory activation, showing significant associations with cardiovascular and neurodegenerative diseases (7). Multiple independent teams have developed disease-specific methylation clocks, including Horvath’s multi-tissue clock (8), Hannum’s blood clock (9), and PhenoAge (10), with predictive efficacy validated across chronic disease cohorts. Emerging prospective studies suggest a potential link between epigenetic age acceleration and stroke risk. Nevertheless, current evidence exhibits substantial heterogeneity: (1) divergent predictive power among methylation clock models; (2) potential confounding from variations in study design, ethnic characteristics, and epigenetic data adjustment strategies. Crucially, no systematic study has clarified whether epigenetic aging influences stroke occurrence independently of traditional vascular risk factors.

To address these gaps, this study employs systematic review and meta-analysis methodologies to synthesize existing evidence, elucidating the association between biological aging and stroke risk. Our findings aim to provide evidence-based insights for early clinical identification and stroke prevention.

## 2 Methods

### 2.1 Search strategy

This meta-analysis and systematic review adhered to the Preferred Reporting Items for Systematic Reviews and Meta-Analyses (PRISMA) guidelines (11) and was prospectively registered in the PROSPERO database (CRD420251010621). A comprehensive literature search was conducted across multiple databases, including EMBASE, PubMed, Web of Science, and the Cochrane Library, with the search period extending through January 10, 2025. No limitations were imposed regarding participants’ age, gender, racial background, or country of origin. To ensure thorough coverage, we additionally examined the reference lists of selected articles and pertinent review papers to identify other potentially relevant studies that might have been missed by our initial search. The complete search methodology is provided in Additional file 1: Supplementary Table S1. Briefly, main search terms included the following terms: (“DNA methylation” OR “methylation” OR “epigenetic” OR biological age) and (“Stroke” OR “Cerebrovascular Accident” OR “Cerebral Stroke” OR “Cerebrovascular Apoplexy” OR “Brain Vascular Accident” OR “Apoplexy”). Two independent investigators (JF and RW), who received standardized training, performed the initial screening of relevant studies. Any discrepancies in their assessments were resolved through discussion until a consensus was reached on the final selection of records.

### 2.2 Inclusion and exclusion criteria

In accordance with the Population, Intervention, Comparison, Outcomes, and Study (PICOS) framework, the inclusion criteria were defined as follows:

(P) The study population was adult participants of all genders and ethnic backgrounds; (I) (Intervention/Exposure) was accelerated biological aging, which was assessed by DNA methylation clocks (e.g., Horvath’s multi-tissue clock, Hannum’s blood clock, PhenoAge) and defined as the deviation between DNA methylation age and chronological age (epigenetic age acceleration, EAA); (C) Controls were participants with non-accelerated biological aging, i.e., those whose DNA methylation age was close to or less than their chronological age; (O) Study outcomes were stroke occurrence, including incident stroke (first-ever stroke) and recurrent stroke, with outcomes quantified by reporting odds ratio (OR), relative risk (RR), or hazard ratio (HR) along with 95% confidence intervals (CIs); (S) Study design included observational studies, specifically prospective cohort studies, retrospective cohort studies, and case–control studies.

The following studies were excluded from the analysis:

(1)  Reviews, letters, personal opinions, book chapters, case reports, conference abstracts, and meeting proceedings; (2) Duplicate publications; (3) Incomplete data; (4) *In vitro* or *in vivo* animal experiments.

### 2.3 Data extraction

Data extraction was performed independently by two authors (JF and XH), who collected study characteristics and relevant outcomes from the selected articles. Initial disagreements in extracted data were resolved through mutual discussion, with any unresolved discrepancies adjudicated by a third reviewer to achieve final consensus. Extracted information included first author (year of publishment), country, participants (including sample size, number of stroke cases, age, and sex distribution), study design, biological sample type used for DNA methylation analysis, epigenetic clock models measured, adjustment for confounders in the statistical analysis, and correlation index of DNA methylation clocks with stroke risk. Studies were permitted to be included multiple times if they reported data from different epigenetic clocks or examined distinct tissue types. For publications containing multiple analytical results, we preferentially selected estimates that accounted for the greatest number of confounding variables to ensure the most robust data extraction.

### 2.4 Assessment of study quality

We referenced Newcastle-Ottawa Scale (NOS) to evaluate the quality of included studies (12). The NOS consisted of 8 items with 9 scores totally, among which assessed the selection (maximum 4 stars), comparability (maximum 2 stars), and outcomes (maximum 3 stars) for cohort study or exposure (maximum 3 stars) for case control study. The Newcastle-Ottawa Scale (NOS) awards a maximum of 9 points, and studies scoring≥ 7 points were deemed high-quality (13) (Additional file 1: Supplementary Table S2).

### 2.5 Statistical analysis

This meta-analysis evaluated the relationship between DNA methylation-based accelerated biological aging and stroke risk using both unadjusted and adjusted OR with corresponding 95% confidence intervals. For studies reporting RR or HR, these measures were analytically treated as equivalent to OR (14). All such effect size estimates were collectively categorized as original data for the purposes of our quantitative synthesis. When OR >1 indicated a closer correlation between DNA methylation-based biological age acceleration and stroke occurrence. The software of Stata 15.0 was applied in this meta-analysis and the heterogeneity among studies was evaluated by Cochran’s *Q* test and *I*^2^ statistics (15). When significant heterogeneity was detected (*I*^2^ ≥ 50% or *p* ≤ 0.05), the random-effects model was employed. Conversely, the fixed-effects model was applied to calculate the observed OR (16). Subsequently, subgroup analyses and meta-regression were conducted to investigate the sources of heterogeneity. Sensitivity analysis was performed using the leave-one-out method, which involves sequentially excluding one study at a time, to assess the robustness of the overall estimates (17). Potential publication bias was evaluated using Begg’s test (18) and Egger’s test (19), funnel plot was also performed to examine publication bias. If publication bias was identified, the trim-and-fill method was utilized to assess the stability of the pooled results (20).

## 3 Results

### 3.1 Characteristics of the included studies

**Figure 1:**
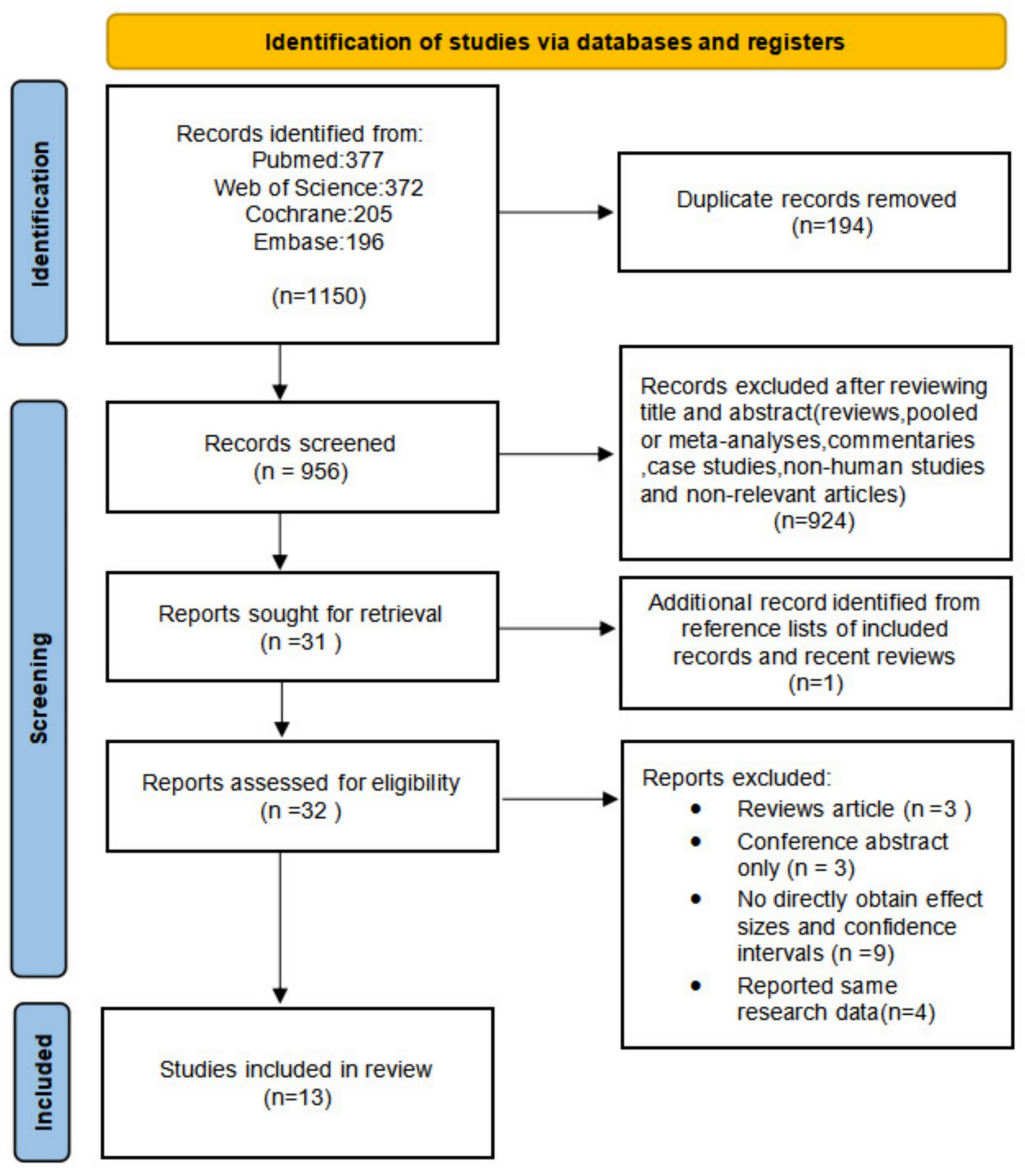
The PRISMA flow diagram showing process of study selection for inclusion in our meta-analyses.

After searching the PubMed, Embase, Cochrane Library, and Web of Science databases, we initially identified 1,150 articles. After removing duplicates (*n* = 194), we screened the remaining records by title and abstract, excluding 924 ineligible studies. The remaining articles (*n* = 32) underwent full-text evaluation. After thorough screening of the study titles, abstracts, and full-texts, a total of 13 articles were included in the final analysis. A flow diagram of the study selection process was shown in Figure 1. Among these 13 articles, 9 were prospective cohort studies (21–29), 3 were retrospective cohort studies (30–32), and 1 was a case–control study (33). Of these studies, 4 were conducted in Asia [2 from Korea (28, 31) and 2 from China (21, 30)], 8 in Europe [3 from the United Kingdom (22, 24, 26), 2 from Spain (32, 33), and 1 each from the Netherlands (23), Germany (25), and Sweden (27)], with 1 study from North America (29). The primary characteristics of included studies were shown in Additional file 1: Supplementary Table S3.

### 3.2 Predictive value of DNA methylation clocks

**Figure 2:**
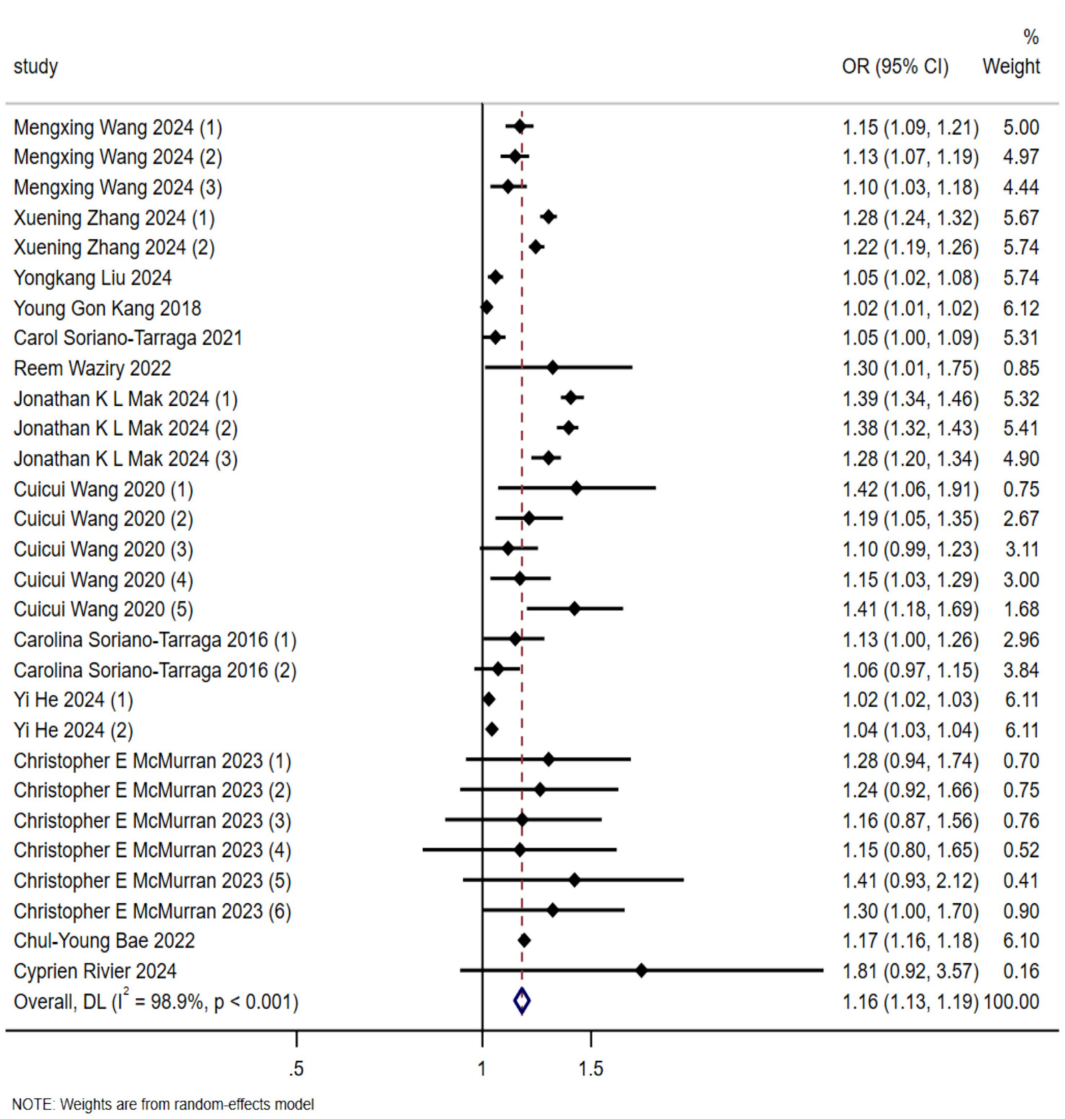
Forest plot of the association between biological aging predicted by DNA methylation clock and stroke occurrence risk (OR, odds ratio; CI, confidential interval).

A total of 29 effect sizes were extracted from the 13 included studies. Figure 2 presents the effect sizes of the association between EAA (measured by DNA methylation clocks) and stroke risk across the included studies, as well as the pooled result. In the forest plot, each row represents an individual study, and the middle section uses squares and horizontal lines to indicate the effect size of each study and its 95% confidence interval (CI), respectively. The pooled analysis revealed a significant association between biologically predicted age acceleration and stroke incidence, with a combined odds ratio of 1.16 (95%CI 1.13–1.19, *p* < 0.001). A random-effects model was employed due to substantial heterogeneity among the studies (*I*^2^ = 98.9%).

### 3.3 Subgroup analyses and meta-regression

To elucidate the sources of heterogeneity, subgroup analyses and univariate meta-regression were conducted on the included effect sizes that were derived from more than three studies. The details could be seen in the Table 1. The results of meta-regression analysis demonstrated that study population (*p* = 0.074), sample size (*p* = 0.866), age (*p* = 0.236), stroke subtype (*p* = 0.279), sex distribution (*p* = 0.264), epigenetic clock type (*p* = 0.773), tissue type (*p* = 0.949), and follow-up duration (*p* = 0.877) were not significant sources of heterogeneity. In contrast, study design (*p* = 0.027) and stroke event classification (*p* = 0.002) were identified as statistically significant sources of heterogeneity for the association between epigenetic age acceleration and stroke risk. However, subsequent subgroup analyses stratified by study design and event classification revealed persistent high heterogeneity within certain subgroups (*I*^2^ > 50%), indicating that these identified factors alone could not fully account for the observed variation. These findings suggest that while the meta-regression detected statistically significant moderators, they likely represent partial rather than exhaustive explanations for heterogeneity. We posit that this residual heterogeneity may stem from both the limited number of included studies (n = 13) and unmeasured confounding variables, particularly baseline population characteristics that were inconsistently reported across studies. Notably, although not reaching statistical significance in our analysis, population demographics (age distribution and ethnic composition) and choice of epigenetic clock methodology emerged as clinically plausible sources of heterogeneity that warrant consideration in future research.

**Table 1:** Subgroup analysis and meta-regression on DNA methylation clock and stroke.

Subgroup	Studies	Pooled OR	Meta-regression	Heterogeneity	
				*I* ^2^	*p*
Population			0.074		
Asian	6	1.1		99.70%	0.001
Others	23	1.19		98.90%	0.001
Study design			0.05		
Prospective cohort	6	1.1		99.70%	0.001
Retrospective cohort	21	1.2		97.50%	0.001
Sample size			0.866		
<5,000	17	1.13		48.60%	0.013
≥5,000	12	1.17		99.60%	0.001
Age (years old)			0.236		
<60	8	1.21		99.60%	0.001
≥60	20	1.13		51.10%	0.005
Stroke type			0.279		
Stroke	15	1.13		99.40%	0.001
Ischemic stroke	14	1.21		94.20%	0.001
Female percentage			0.264		
<50	12	1.14		99.40%	0.001
≥50	17	1.19		97.90%	0.001
Clock type			0.773		
PhenoAge	8	1.17		97.90%	0.001
KDMAge	5	1.2		99%	0.001
Tissue			0.949		
Blood	16	1.18		95.70%	0.001
Multi-tissue	6	1.18		98.70%	0.001
Event			0.002		
Recurrence	4	1.11		63.60%	0.041
Incidence	13	1.28		92.60%	0.001
Follow up years			0.877		
<10	9	1.19		98.50%	0.001
≥10	12	1.16		99.20%	0.001

*I*^2^ > 50% with the random effects model; *I*^2^ < 50% with the fixed-effects model; OR, odds ratio.

### 3.4 Publication bias and sensitivity analysis

**Figure 3:**
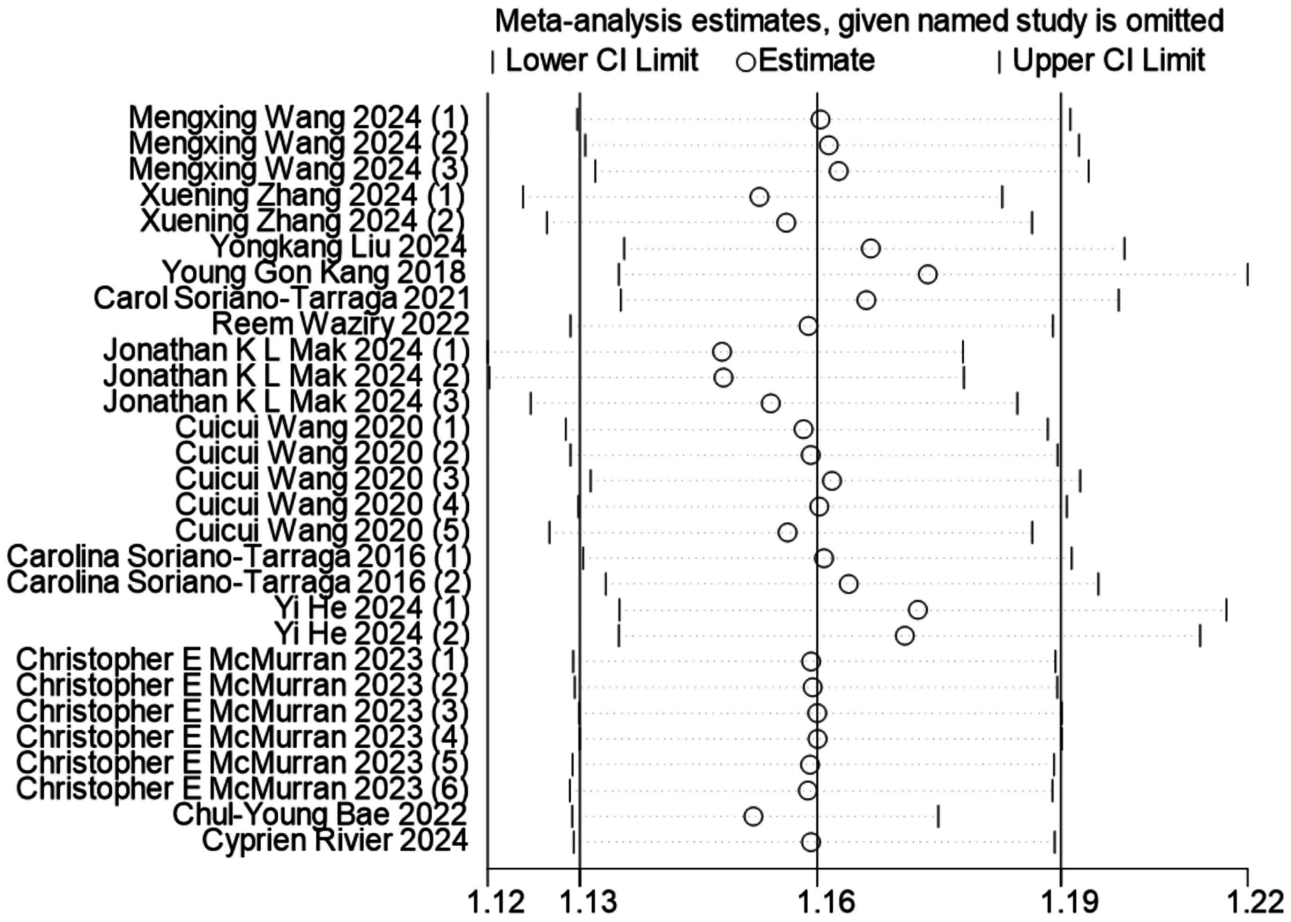
Sensitivity analysis of the association between biological age predicted by DNA methylation clock and stroke occurrence risk.

Publication bias was assessed through visual inspection of funnel plot symmetry and formal statistical testing using Begg’s and Egger’s tests. Visual inspection of the funnel plot suggested asymmetry, indicating potential publication bias (Additional file 2: Supplementary Figure S1). In our meta-analysis, Begg’s test indicated no significant publication bias (*p* = 0.053) (Additional file 2: Supplementary Figure S2), whereas Egger’s test revealed evidence of potential publication bias in the estimates of the biological aging-stroke association (*p* = 0.006) (Additional file 2: Supplementary Figure S3). Following adjustment for funnel plot asymmetry using the trim-and-fill method, the significant association between DNA methylation-based age acceleration and stroke risk remained robust (Additional file 2: Supplementary Figure S4). Sensitivity analysis demonstrated the stability of our findings, as shown in Figure 3, where the sequential exclusion of individual studies did not substantially alter the overall effect estimate, confirming the reliability of our meta-analytic results.

## 4 Discussion

This study, through a systematic review and meta-analysis, elucidates the association between DNA methylation-derived accelerated biological aging and stroke risk, addressing prior inconclusive evidence and the lack of comprehensive synthesis in this area. The results confirm a significant positive association between EAA and stroke incidence (OR = 1.16, 95% CI 1.13–1.19), with a stronger correlation observed in incident stroke (OR = 1.28, 95% CI 1.25–1.35) compared to recurrent stroke (OR = 1.11, 95% CI 1.06–1.16). This finding suggests that EAA could serve as a novel biomarker for stroke risk assessment, particularly for identifying individuals at risk of a first-ever stroke.

Increasing evidence suggests that epigenetic age, as measured through DNA methylation clocks, is a more reliable biomarker of biological aging and disease susceptibility than chronological age (34, 35), thus supporting our findings on EAA and stroke. As previously noted, advanced epigenetic clocks (e.g., DNAm PhenoAge, DNAm GrimAge) integrate clinical biomarkers and methylation signatures linked to lifestyle factors, thus enhancing predictive accuracy for various conditions, including cardiovascular diseases, cancer, and neurodegenerative disorders (10). This predictive capacity also extends to stroke-related outcomes: a 10-year longitudinal study found that accelerated biological aging is associated with a higher mortality risk among stroke patients (HR = 1.33, 95% CI 1.26–1.41) (36), emphasizing the relevance of epigenetic aging not only to stroke incidence but also to post-stroke prognosis.

Notably, our subgroup analysis revealed that study design (*p* = 0.027) and stroke event classification (*p* = 0.002) were significant sources of heterogeneity, while clock type (*p* = 0.773) was not, despite the biological differences between clocks. For example, PhenoAge, which integrates clinical biomarkers (e.g., albumin, C-reactive protein) (10), might be more closely tied to vascular physiological aging than Horvath’s multi-tissue clock, yet our analysis did not detect a significant difference in effect size. This may be due to the small number of studies using each clock, which limits statistical power to detect clock-specific effects—a gap that future studies with larger clock-stratified samples should address.

To explain the association between EAA and stroke risk, we draw on plausible biological mechanisms supported by existing evidence and our findings. First, vascular aging, a key driver of stroke, is tightly regulated by DNA methylation. Genes such as ELOVL2 (fatty acid elongase) and KLF14 (transcription factor) (37), whose methylation status defines EAA, are essential for vascular elasticity and endothelial function. Accelerated methylation of these genes suppresses their expression, promoting arteriosclerosis and endothelial dysfunction. These processes are more evident in first-ever stroke (OR = 1.28), which reflects the greater impact of cumulative vascular damage from biological aging. Second, chronic inflammation—a hallmark of both aging and stroke (38)—is amplified by EAA. Hypomethylation of pro-inflammatory cytokines (IL-6, TNF-α) (39, 40) sustains inflammation, damages the vascular endothelium, and accelerates atherosclerosis. In recurrent stroke, where vascular damage is already established, the additional contribution of EAA and its related inflammation may be smaller, consistent with the lower OR of 1.11. Third, impairment of the neurovascular unit (NVU)—a central pathological feature of stroke—can be exacerbated by EAA. Increased methylation of SIRT1 (a neuroprotective gene) (41) suppresses its expression, weakens the blood–brain barrier (BBB), and heightens brain vulnerability to ischemic injury. Collectively, these mechanisms indicate that EAA functions as a “biological integrator” of vascular, inflammatory, and neural damage, thereby contributing to stroke risk in a subtype-specific manner.

Despite support from the aforementioned mechanisms, the clinical application of EAA warrants cautious consideration, particularly because of its modest effect size (OR = 1.16). Compared with established stroke risk factors (e.g., hypertension; diabetes mellitu), the independent contribution of EAA is substantially lower. Therefore, EAA cannot replace traditional risk assessment tools such as the CHA₂DS₂-VASc score. Nevertheless, EAA holds potential as a complementary biomarker. It can provide biological information not captured by traditional factors, aid in optimizing risk stratification, and offer additional support for clinical decision-making. The core challenges in translating EAA into clinical practice currently include two aspects. First, there is no international consensus on defining the quantitative threshold for “high-risk EAA” (i.e., the number of years of accelerated biological aging that can be deemed as an elevation in stroke risk). Second, existing studies have only confirmed the association between EAA and stroke risk, but have not verified whether intervening in EAA (e.g., regulating relevant methylation sites) can reduce the incidence of stroke.

To enhance the translational value of DNA methylation–based EAA in stroke risk assessment, future research should prioritize standardized methodological frameworks. These include adopting validated, vascular health–specific epigenetic clocks and using uniform stroke outcome definitions—explicitly excluding transient ischemic attack and standardizing subtype classification—to minimize heterogeneity. At the same time, cohort diversity should be expanded to include underrepresented populations from Africa, South America, and Oceania, enabling race-stratified analyses that clarify ethnic specificity in the EAA–stroke association. In addition, future studies should aim to establish causality through Mendelian randomization, using EAA-related genetic variants as instrumental variables. They should also evaluate interventional efficacy in trials targeting EAA (e.g., lifestyle modification or epigenetic regulators), and integrate EAA into traditional risk models to assess incremental predictive value using metrics such as AUC and net reclassification improvement. Finally, defining quantifiable “high-risk EAA” thresholds through dose–response meta-analyses or machine learning, and validating these across diverse populations, will be essential to advancing EAA from a biological marker to a clinically actionable tool in stroke prevention.

One of the strengths of this study lies in its pioneering nature: to our knowledge, this is the first study to conduct a systematic review and meta-analysis of the association between DNA methylation clocks and stroke risk, representing a comprehensive approach that, even if not exhaustive, can better elucidate the relationship between this potentially promising biomarker and stroke. Additionally, all studies included in this analysis were of high quality, with scores above 7 on the NOS, thereby ensuring reliability. All articles included in this analysis were published within the past decade, reflecting the latest research in this field.

Despite these strengths, several limitations of this study must be acknowledged. First, substantial heterogeneity remained across studies even after subgroup analysis (*I*^2^ = 98.9%). This may be explained by unmeasured factors such as inconsistent confounder adjustment, variation in tissue types used for methylation detection, and unreported baseline characteristics (e.g., socioeconomic status, dietary patterns). Second, Egger’s test indicated publication bias (*p* = 0.006), which may reduce the reliability of the pooled effect size. Although we applied trim-and-fill adjustment to reduce this bias, residual distortion cannot be excluded, especially given the small number of included studies (*n* = 13), which limits the effectiveness of correction methods. Third, small-sample bias may have influenced the results. Six of the 13 studies included fewer than 5,000 participants, and these smaller studies tended to report more variable effect sizes, potentially amplifying the observed heterogeneity. Fourth, the lack of individual-level data prevented adjustment for detailed confounders (e.g., baseline BMI, insulin resistance, smoking intensity). These factors may correlate with both EAA and stroke risk, introducing residual confounding that could bias the observed association. Fifth, insufficient data prevented evaluation of differences in the predictive ability of specific clocks for stroke risk.

## 5 Conclusion

This systematic review and meta-analysis demonstrates that DNA methylation clocks represent a potentially promising tool for stroke risk prediction. However, further research is needed to better identify stratification algorithms and develop specific epigenetic clocks and measurements, which must be validated across diverse populations. The growing body of literature on epigenetic measures calls for systematic organization and classification to reduce heterogeneity between studies and facilitate the translation of these tools into clinical practice and preventive strategies. In the future, personalized aging assessment systems based on DNA methylation clocks may emerge as a critical breakthrough in precision medicine for cerebrovascular diseases.

## Data Availability

The original contributions presented in the study are included in the article/Supplementary material, further inquiries can be directed to the corresponding author.
