# Supplementary material for: Accelerated biological aging based on DNA methylation clocks is a predictor of stroke occurrence: a systematic review and meta-analysis
Source: Front Neurol. 2025 Nov 6;16:1640853. doi: 10.3389/fneur.2025.1640853 (PMC12631622; doi:10.3389/fneur.2025.1640853)
Supplement: Supplementary file 2 [file Data_Sheet_1.docx]

**
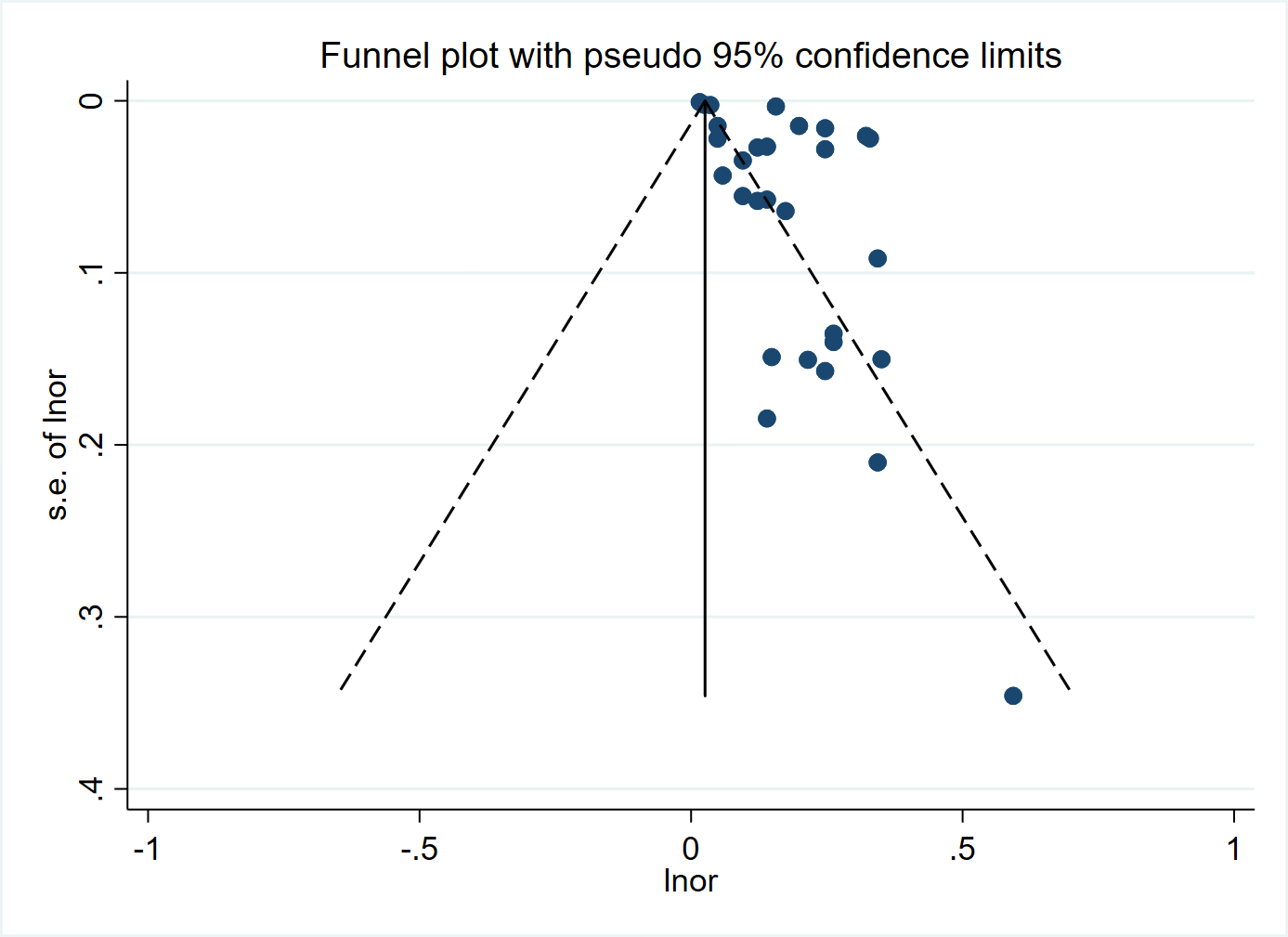
**

**Figure S1. Funnel plot for studies (of 13 studies) on the association between accelerated biological aging based on DNA methylation clocks and stroke occurrence.**

**Figure S2. Begg’s funnel plot**

**Figure S3. Egger’s funnel plot**

**Figure S4. Adjusted funnel plot.**
